# Clinical significance in COPD patients followed in a real practice

**DOI:** 10.1186/2049-6958-8-43

**Published:** 2013-06-28

**Authors:** Júlio César Mendes de Oliveira, Isabella de Carvalho Aguiar, Ana Carolina Negrinho de Oliveira Beloto, Israel Reis Santos, Fernando Sergio Studart Leitão Filho, Luciana M Malosa Sampaio, Claudio F Donner, Luís Vicente Franco de Oliveira

**Affiliations:** 1 Institute of the Lung, Cascavel, PR, Brazil; 2 Rehabilitation Sciences Master and Doctoral Degree Program - Nove de Julho University, UNINOVE, Sao Paulo, SP, Brazil; 3 Respiratory Division of Medicine School, Fortaleza University, UNIFOR, Fortaleza, CE, Brazil; 4 Mondo Medico, Multidisciplinary and Rehabilitation Outpatient Clinic, Borgomanero, No, Italy

**Keywords:** COPD, Dyspnea, Smoking

## Abstract

**Background:**

Chronic obstructive pulmonary disease (COPD) is an important public health issue in many countries which is estimated to become the fifth cause of disability and the third cause of mortality in the world within 2020.

The objective of this study was to identify the clinical characteristics in the real clinical practice of a sample of patients with COPD followed in a pulmonology clinic.

**Methods:**

The initial sample contained 207 subjects with respiratory claims that searched for specialized treatment and initiated regular monitoring between 2004 and 2009 in a private clinic localized in Cascavel, in the state of Parana, Brazil. Demographic data (weight, height, body mass index - BMI), history of comorbidities, use of respiratory and non respiratory drugs were also registered.

**Results:**

The main cause related to the development of COPD was current or prior smoking (92.0%); the most frequently reported symptom was dyspnea (95.0%), followed by cough (86.1%), wheezing (69.4%) and sputum production (40.0%). During the follow up, 51 patients developed the need for oxygen therapy (28.3%). In 96 patients, there were periods of acute exacerbation, resulting in 37 hospitalizations. In addition to COPD, a significant number of comorbidities were identified, being cardiovascular disease and neurological disorders the most prevalent ones.

**Conclusions:**

Based on the data collected, we could outline the profile of patients with COPD, showing characteristics of an elderly population, with multiple comorbidities, suggesting a health related quality of life lower than expected.

## Background

Chronic obstructive pulmonary disease (COPD) is an important public health issue in many countries which is estimated to become the fifth cause of disability and the third cause of mortality in the world within 2020 [1,2].

The prevalence of COPD in the world’s population is considered to be around 1% concerning all age groups, rising to 8-10% or more in individuals ≥ 40 years old [3,4]. In Brazil, the PLATINO study (Latin American Project for the Investigation of Pulmonary Obstruction) identified a COPD prevalence of 15.8% in the population living in the metropolitan region of São Paulo, while another epidemiological study demonstrated that the prevalence of COPD is 9.1% when considering the population between 40 and 70 years old [6].

Few studies have provided data about the epidemiology of COPD [5,7-9] in the Latin America. Exposition to respiratory risk factors also differ from one geographical area to another [10] and these factors, associated to genetic predisposition and smoking, may be the reason for the different features and severities of COPD patients among the countries [11]. In recent years, epidemiological studies have demonstrated a high and rising cost of COPD around the world [3,4]. Moreover, the adherence to treatment guidelines is low, especially in the less developed countries [12-15].

COPD patients followed up in outpatients clinics are usually elderly and have, along with the respiratory disease, many other comorbidities [16,17]. There are thousands of articles in the literature devoted specifically to patients with COPD, however, as any clinical study has specific inclusion and exclusion criteria, the current researches are restricted to subgroups of COPD patients, making a substantial number of other patients ineligible primarily because of their comorbidities. In this way, it is increasingly more difficult to delineate the true profile of COPD patients in outpatient settings [10]. This paper aims at characterizing these patients from the assessment of demographic, clinical, and spirometric variables.

## Methods

In this cross-sectional study, the initial sample was composed of 207 subjects with respiratory complaints who looked for specialized pneumological care and started a regular follow up between 2004 and 2009 in a private clinic in Cascavel, in province of Parana, Brazil.

The diagnosis of COPD was confirmed by compatible clinical presentation, presence of known risk factors as smoking or exposition to biomass combustion (especially that from burned-over land) and observation of post-bronchodilator FEV_1_/FVC ratio < 0.70 at spirometry, according to Global Initiative for Chronic Obstructive Lung Disease (GOLD) guidelines [2,16,17].

During the evaluation visit, all patients underwent detailed anamnesis and physical examination, both conducted by a pneumologist. It was investigated: the patient’s main symptoms; degree of basal dyspnea according to the modified Medical Research Council scale (mMRC) [18]; causes related to COPD development (smoking or exposition to biomass combustion); previous or current tobacco consumption; measurement of tobacco exposure in pack/years; previous number of hospitalization events and exacerbations; current use of long-term oxygen therapy (LTOT); search for non-respiratory comorbidities and description of all respiratory and non-respiratory medications currently in use.

In the same day of clinical evaluation, study subjects’ weight and height were measured. These subjects underwent pre and post-bronchodilator spirometry, following administration of albuterol 400 μg via MDI device, according to the American Thoracic Society (ATS) guidelines for lung function [19]. Patients were previously informed by phone to discontinue bronchodilator use before spirometric evaluation, so that short or long acting β_2_-agonists and tiotropium were withheld for at least 6, 12 and 24 h, respectively.

There was no restriction on the number or type of inhaled or oral medications prescribed by the pneumologist for controlling respiratory symptoms; they varied according to clinical and spirometric data, as well as each patient’s income.

This study was approved by the Committee of Ethics and Research of UniversidadeNove de Julho (UNINOVE–Brazil) under Protocol 219561/2008. All patients signed Informed Consent prior to participation and had the option to withdraw from the study at any time.

### Statistical analysis

The data are presented in a descriptive mode. The variables, when indicated, are presented in absolute numbers, average, and standard deviation or as percentages.

## Results

Our sample involved 180 patients with respiratory complaints, with an average age of 67.7 ± 10.1 years. There was an absolute prevalence of male gender (79%). The main cause related to COPD development was previous or current tobacco consumption (92%), including 9 cases probably related to passive tobacco smoke exposure (5%). The average tobacco smoke exposure was 51 ± 14.9 pack/years. Another less frequent cause was the exposure to biomass combustion (wood-fired ovens and burned-over land), identified in 17 patients (9.4%). The average body-mass index (BMI) was 24.9 ± 5.2 kg/m^2^ with 45 patients (25%) presenting a BMI ≥ 21 kg/m^2^ (Table 1).

**Table 1:** Baseline demographics of study population (n = 180)

**Variables**	**Values**
**Age, years**	**67.7 ± 10.1**
**Sex (M:F), N (%)**	**142 (79%)/38 (21%)**
**Weight, Kg**	**69.1 ± 16.4**
**BMI, kg/m**^**2**^	**24.9 ± 5.2**
**Smokers (current and former), N (%)**	**166 (92.0%)**
**Smoking, pack/years**	**51 ± 14.9**
**Biomass exposure, N (%)**	**17 (9.4%)**

Data are expressed as means ± SD unless otherwise indicated.

The most frequently reported symptom was dyspnea (95%) and 72.4% of the patients referred to dyspnea degree 3 or 4 according to the mMRC scale. The other symptoms, sorted in decreasing frequency order, were chronic cough (86.1%), wheezing (69.4%), and sputum production (40%). The average post-bronchodilator FEV_1_ was 44.8 ± 16.7% of predicted, with most patients being classified into GOLD III (36.7%) and IV (20.5%) stages. During the follow up, 51 patients evolved with need for LTOT (28.3%); among these patients in 13 cases (25%), after the optimization of bronchodilator treatment, there was discontinuation of oxygen supplementation. Acute periods of exacerbation occurred in 96 subjects (53.3%), eventually amounting to 119 episodes and resulting in 37 hospitalizations. Table 2 describes the main clinical features of the evaluated patients.

**Table 2:** Clinical and functional characteristics of evaluated COPD patients (n = 180)

**Dyspnea**	**171 (95%)**
**Dyspnea Degree 1 (mMRC)**	**16 (8.8%)**
**Dyspnea Degree 2 (mMRC)**	**25 (13.8%)**
**Dyspnea Degree 3 (mMRC)**	**92 (51.1%)**
**Dyspnea Degree 4 (mMRC)**	**38 (21.3%)**
**Chronic Cough**	**155 (86.1%)**
**Sputum Production**	**72 (40.0%)**
**Wheezing**	**125 (69.4%)**
**Post-bronchodilator Spirometry:**	**Mean ± SD**
**FVC (L)**	**2.19 ± 0.99**
**FVC (% of predicted)**	**75.0 ± 15.0**
**FEV**_**1**_**(L)**	**1.17 ± 0.52**
**FEV**_**1**_**(% of predicted)**	**44.81 ± 16.7**
**FEV**_**1**_**/FVC (%)**	**57 ± 12**
**COPD stage:**	**N (%)**
**GOLD I**	**14 (7.8%)**
**GOLD II**	**63 (35.0%)**
**GOLD III**	**66 (36.7%)**
**OLD IV**	**37 (20.5%)**

Data are expressed as number (%) or means ± SD as appropriate.

**Figure 1:**
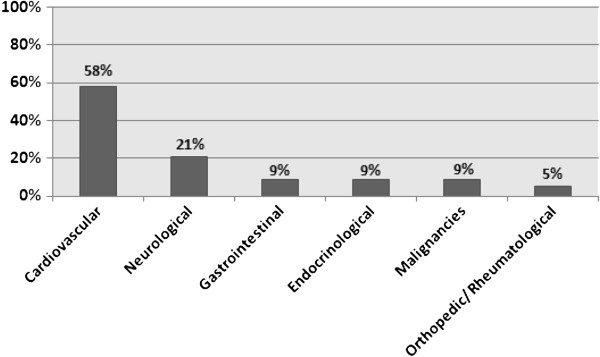
Comorbidities found in evaluated COPD patients.

Evaluating the clinical history of follow up cases, we identified, along with COPD, a significant number of comorbidities, being the most prevalent the cardiovascular and neurological diseases, observed, respectively, in 58% and 21% of the subjects (Figure 1). Hypertension was the most common cardiovascular comorbidity, identified in 100 patients (55%). In 79 patients (43.9%) only a non-respiratory comorbidity was diagnosed, while 39 patients (21.7%) had two comorbidities and 12 cases (6.7%) presented three or more comorbidities. In face of it, 90 patients (43.4%) reported daily use of three medications (including the respiratory ones), while 70 (33.8%) use four medications and 47 (22.8%) use five or more medications. Table 3 displays the main classes of respiratory and non-respiratory medications used by the participants in the sample.

**Table 3:** Use of respiratory and non-respiratory medications among evaluated COPD patients (n = 180)

**Non-respiratory medications**	**N (%)**
**Antihypertensivesincluding diuretics**	**155 (86.1%)**
**Antidiabetics**	**26 (14.5%)**
**Platelet antiaggregants**	**37 (20.5%)**
**Antidepressants**	**30 (16.6%)**
**Analgesics**	**7 (3.8%)**
**Digitalics**	**52 (28.8%)**
**Prokinetics**	**17 (9.4%)**
**Arterial vasodilators**	**32 (17.7%)**
**Antiarrhythmics**	**35 (19.4%)**
**Hypolipidemic drugs**	**39 (21.6%)**
**Respiratory medications**	**N (%)**
**Short-acting β**_**2**_**-agonists/Ipratropium**	**160 (88.9%)**
**Long-acting β**_**2**_**-agonists**	**14 (7.8%)**
**Long-acting β**_**2**_**-agonists plus inhaled corticosteroids**	**162 (90%)**
**Tiotropium**	**57 (31.7%)**
**Inhaled corticosteroids**	**5 (2.8%)**
**Methilxantines**	**153 (85%)**

In our study, the symptoms of COPD were more frequently ameliorated by prescription of long-acting β_2_-agonists (formoterol or salmeterol) combined to inhaled corticosteroids (90%) as a maintenance treatment. The tiotropium was prescribed to 31.7% of the patients, especially in case of persistence of symptoms among patients already taking other classes of bronchodilators. As relief medication, the most used drugs were short-acting β_2_-agonists (albuterol or fenoterol) (88.9%). Another interesting fact of our study concerned the analysis of non-respiratory medications, with 86.1% of patients taking regularly antihypertensives and diuretics (86.1%), for example. Table 3 displays the main respiratory and non-respiratory medications regularly used in the studied sample.

There was no difference in the frequency of exacerbations, use of LTOT and comorbidities according to gender. However, we observed a higher frequency of comorbidities in patients >60 years old in comparison to patients ≤60 years, and this finding achieved statistical significance (p = 0.04).

## Discussion

The profile of COPD patients of our study revealed that they are: elderly, mainly men, usually in advanced stages of the disease and presenting multiple comorbidities. Smoking was related by 92% of patients, being probably the most common factor associated with COPD development in this study, what is in accordance to the available literature [2,5].

Higher age is compatible with the period between the beginning of exposure (usually tobacco smoke) and the development of COPD, as less aged patients reflect probably a higher genetic predisposition or even deficiency of α1-antitrypsin [2,20]. The prevalence of male gender is consistent with previous studies in literature, with one study indicating the presence of 83% male individuals among more than 10,000 patients followed up in a respiratory clinic in Spain [17,21]. This finding probably reflects the higher prevalence of tobacco smoking and a higher exposure to occupational activities such as biomass combustion in male gender, which could explain the higher incidence of COPD among men [10,22,23]. Because of higher age, the presence of other non-respiratory comorbidities is more common in COPD patients; in our sample we observed that 72.2% of the subjects had at least one non-respiratory comorbidity, with cardiovascular diseases (coronary insufficiency, heart failure and arrhythmia) being the most prevalent ones. In the multicentric EPOCA (Enfermedad Pulmonar Obstructiva Crónica en Acción) Project, which was also dedicated to the analysis of the features of patients with COPD, the prevalence of comorbidities varied considerably according to the evaluated country, oscillating from 38.1% in Argentina to 63.4% in Spain [17].

The main symptom reported by the population studied was dyspnea (92%), reflex of the ventilatory limitation observed in COPD. The EPOCA study found similar results, with dyspnea being reported by 97% of the patients, followed by chronic cough (79.6%) and sputum production (70.5%) [17]. COPD stages II and III were the most prevalent (71.7%); this probably may be explained by delayed diagnosis (sub-diagnosis) at the earlier stages, since many patients initially ascribe their effort intolerance, due to COPD, to aging and sedentarism. The PLATINO study identified that 85.7% of the 144 patients diagnosed with COPD in the metropolitan region of Sao Paulo had never received this diagnosis in their lives [24]. Moreover, the same study observed that, among the patients previously diagnosed with COPD, 42.7% had never been counseled to stop smoking and 82.3% were not receiving the recommended pharmacologic treatment.

Regarding the use of medications for the respiratory system, it is interesting to note that almost 60% of the sample consisted of patients with stage III and IV of the GOLD classification. Based on this finding, the high prescription in our sample of patients of β_2_-agonists associated with long-term inhaled corticosteroids (97.8%) and tiotropium (31.7%) is easily explicable.

Similarly, also the great use of methylxanthines among patients (85%) can be explained. The GOLD guidelines point out that inhaled bronchodilators, where available, should be used as first-line treatment in COPD, rather than oral bronchodilators, including methylxanthines. However, the same guidelines emphasize that methylxanthines can and should be considered in the treatment of patients with COPD who remain symptomatic despite the use of inhaled bronchodilators such as long-acting β_2_-agonists association and tiotropium. Furthermore, methylxanthines are low cost medications in Brazil, compared to some classes of inhaled bronchodilators, which may have contributed to the prescription above expectations, according to recommendations from international guidelines.

The presence of LTOT in our sample is consistent with previous studies, which reported that around 25% of the followed subjects with COPD use LTOT [13,21,25]. The high frequency of LTOT among these patients is justified by the loss in lung function and the ventilation/perfusion mismatch, especially in the most advanced phases of COPD [2,13].

We observed that almost one-third of the patients use digitalic drugs daily; this is probably related to left ventricle systolic dysfunction (LVSD) or even right failure due to corpulmonale. A recent systematic review revealed that the connection between COPD and LVSD is probably much more prevalent than previously estimated; superimposition rates between these two diseases varied from 10% to 46%, especially during periods of COPD exacerbation [26]. In our sample, corpulmonale was identified in 30 patients (16.6%), whereas the presence of LVSD was suggested in 20 patients (11.1%), according to echocardiographic findings.

We also point out the high incidence of systemic arterial hypertension (55%) in the evaluated subjects (average age 67.7 years), since the prevalence of this comorbidity (55%) increases linearly with age [26,27]. Besides that, many patients were using diuretics specifically to ameliorate edema due to corpulmonale, or angiotensin-converting enzyme (ACE) inhibitors due to LSVD, thus explaining the use of antihypertensives (including diuretics) in 155 patients (86.1%).

It is important to emphasize that almost 20% of the patients took regularly antidepressants, a possible direct reflex of patients’ compromised health status and quality of life [23]. In this context, it is interesting to quote a recent case–control study with more than 35,000 patients with COPD which identified that the existence of COPD itself doubles the risk of depression (OR 2.01, IC95% 1.45-2.78) [28]. Another finding of concern in our study is the fact that the evaluated patients took many different types of drugs, an average of at least four medications a day. This is clinically relevant, since COPD patients are usually elderly patients, who frequently experience visual, hearing, and even cognitive deficits, which could possibly reduce their compliance to these medications, especially when they are not adequately assisted by their relatives [29].

## Conclusion

In conclusion, we observed that COPD patients followed up are usually elderly, mainly men, and have multiple comorbidities, especially cardiovascular diseases. Therefore, a good design of the clinical profile of patients with COPD may probably result in a more effective and individualized therapeutic approach. As limitation of our study we can quote the fact we did not evaluate the impact of respiratory and non-respiratory comorbidities in the quality of life, as well as the compliance to medications in use.

## Competing interests

The authors declare that there is no association with any commercial enterprise that has interest in the object of this study.
